# An unusual two-dimensional MOF formed from Ni^II^, thio­phene-2,5-di­carboxyl­ate and *trans*-1,2-bis­(pyridin-4-yl)ethyl­ene

**DOI:** 10.1107/S2056989026002276

**Published:** 2026-03-05

**Authors:** Chongting Ren, Xu Jia, Luc Van Meervelt

**Affiliations:** a Department of Chemistry, KU Leuven, Biomolecular Architecture, Celestijnenlaan 200F, Leuven (Heverlee), B-3001, Belgium; University of Buenos Aires, Argentina

**Keywords:** crystal structure, metal-organic framework, nickel, thio­phene-2,5-di­carboxyl­ate, *trans*-1,2-bis­(pyridin-4-yl)ethyl­ene

## Abstract

A new Ni^II^ MOF, poly[[sesqui[μ-*trans*-1,2-bis­(pyridin-4-yl)ethyl­ene](μ-thio­phene-2,5-di­carboxyl­ato)nickel(II)] di­methyl­formamide 0.205-solvate], {[Ni(C_6_H_3_O_4_S)(C_12_H_10_N_2_)_1.5_].0.205C_3_H_7_NO}_*n*_, was obtained under solvothermal conditions and its structure was determined by single-crystal X-ray diffraction. The structure reveals that Ni nodes are bridged by thio­phene-2,5-di­carboxyl­ate (HT) and *trans*-1,2-bis­(pyridin-4-yl)ethyl­ene (Bpe) to generate an unusual two-dimensional layered framework, and the overall crystal is formed by an inter­locked stacking of these layers. Topological simplification classifies the framework as a non-inter­penetrated 3-nodal (2,2,5)-connected net, in which the Ni-containing node acts as the higher-connected vertex and the two organic ligands serve as 2-connected linkers propagating the connectivity within the layer. The experimental powder X-ray diffraction (PXRD) pattern is in good agreement with that simulated from the single-crystal structure, further confirming that the powder sample is consistent with the single-crystal model and exhibits good phase purity.

## Abstract 2

A new Ni^II^ MOF, C_24_H_17_N_3_NiO_4_S·0.205(C_3_H_7_NO), was obtained under solvothermal conditions and its structure was determined by single-crystal X-ray diffraction.

## 1. Chemical context

Metal–organic frameworks (MOFs) continue to attract strong inter­est in crystal engineering owing to their high structural tunability and rich topological diversity (Furukawa *et al.*, 2013 ▸). This diversity arises primarily from the wide range of coordination numbers and geometries accessible to metal nodes (or clusters), together with the adjustable connectivity, length and conformation of organic linkers (O’Keeffe & Yaghi, 2012 ▸). As a result, MOFs can display varied dimensionalities and connectivity patterns, often accompanied by key structural features such as porosity, layered packing and inter­penetration. In this context, mixed-ligand strategies provide an efficient route to modulate connectivity and spatial extension by combining complementary coordinating groups, thereby expanding the diversity and accessibility of framework architectures and underlying topologies (Yin *et al.*, 2015 ▸).

Within the widely used combination of di­carboxyl­ate and N-donor linker construction method, thio­phene-2,5-di­carboxyl­ate (HT) exhibits distinctive features. The thio­phene core introduces a sulfur-containing heteroaromatic, π-conjugated motif, so that, in addition to providing robust carboxyl­ate bridges, it may influence inter­layer packing and framework dimensionality through π–π inter­actions and other weak supra­molecular contacts (Thuéry & Harrowfield, 2022 ▸; Zheng *et al.*, 2008 ▸). Meanwhile, the linear N-donor ligand *trans*-1,2-bis­(pyridin-4-yl)ethyl­ene (Bpe) is rigid and offers a relatively long spacer length; its terminal pyridyl N atoms impart well-defined directional coordination and it is therefore frequently employed as a ‘pillar’ to tune metal–metal separations, promote layered architectures, and regulate the underlying topology (Wu *et al.*, 2019 ▸; Zhang *et al.*, 2012 ▸). Accordingly, the synergistic assembly of HT and Bpe provides a suitable platform for constructing frameworks with characteristic layered motifs and topological features.

On this basis, a new Ni^II^ MOF, Ni-HT-Bpe, was obtained under solvothermal conditions and its structure was determined by single-crystal X-ray diffraction. Notably, the framework adopts a thick parallel polycatenated 2D entangled architecture, which belongs to a comparatively rare subclass among entangled 2D coordination networks (*ca*. 9.2% overall in an extended ring net (ERN)-based statistical survey; Alexandrov *et al.*, 2017 ▸). Moreover, closely related systems based on the same (or very similar) linker combinations more commonly form twofold inter­penetrated 3D frameworks (Jia *et al.*, 2024 ▸; Alamgir *et al.*, 2021 ▸; Sen *et al.*, 2013 ▸), highlighting an unusual structure–composition relationship for Ni-HT-Bpe.
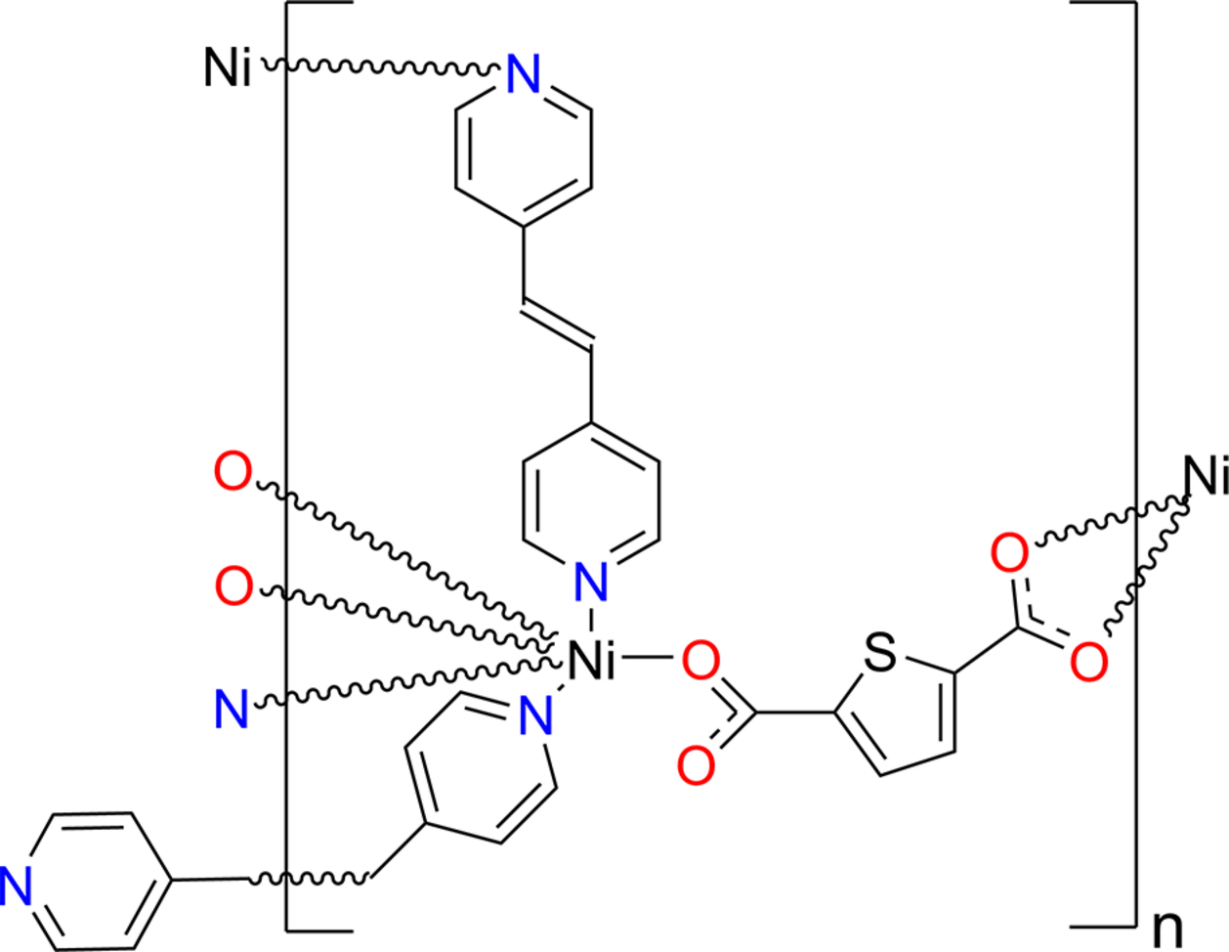


## 2. Structural commentary

**Figure 1:**
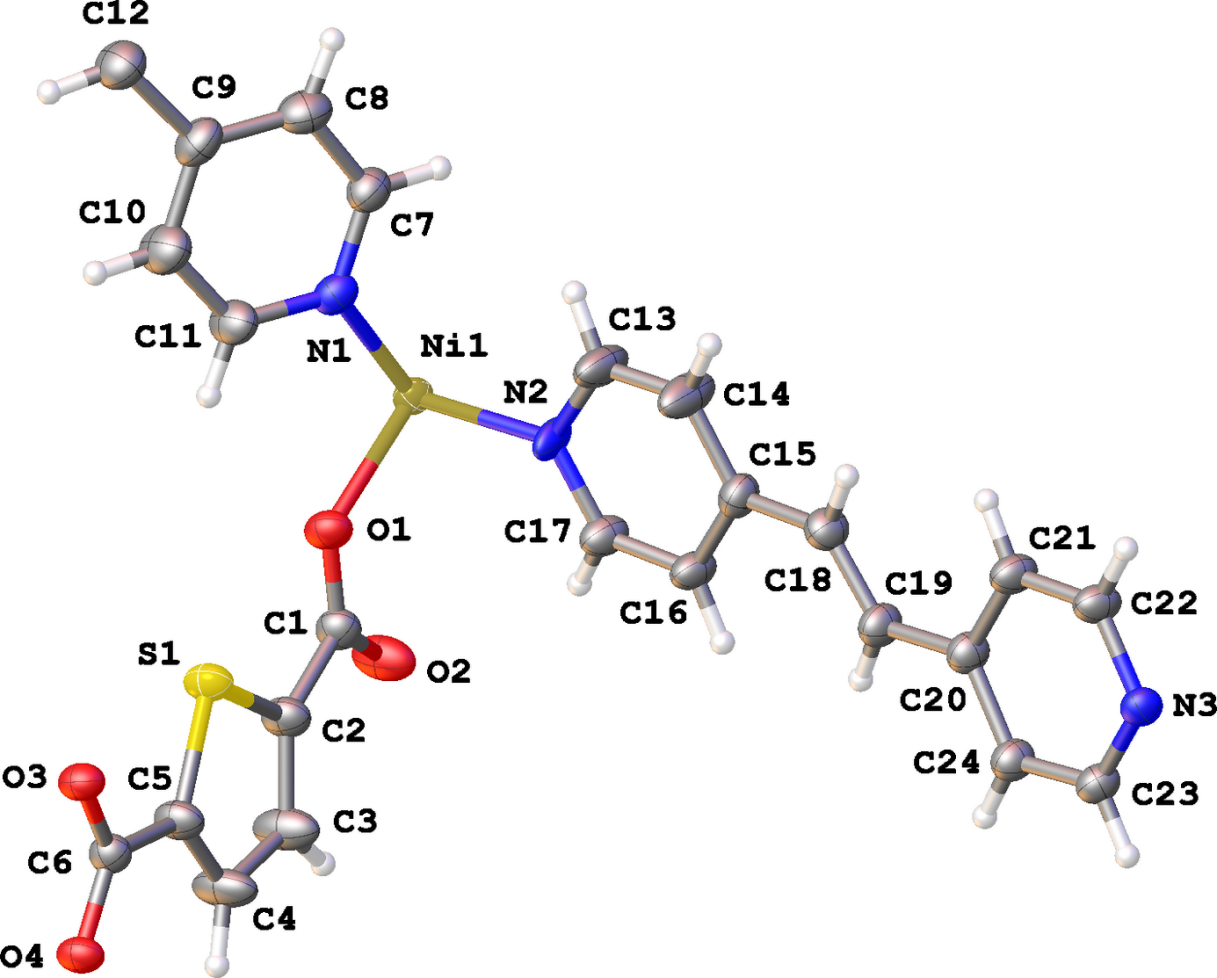
Asymmetric unit of the title compound Ni-HT-Bpe showing the atom labelling and 30% probability ellipsoids. Only the major component of the disordered *trans*-1,2-bis­(pyridin-4-yl)ethyl­ene is shown and the partial di­methyl­formamide mol­ecule has been removed for clarity.

The Ni-HT-Bpe structure crystallizes in the triclinic space group *P*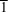 with one Ni ion, one thio­phene-2,5-di­carboxyl­ate and one and a half *trans*-1,2-bis­(pyridin-4-yl)ethyl­ene in the asymmetric unit (Fig. 1 ▸). The asymmetric unit also contains a di­methyl­formamide (DMF) mol­ecule with an occupancy of 0.205 (7) close to the inversion center at 1/2,1/2,1/2 generating a second DMF. The second Bpe half is generated by inversion symmetry. The other Bpe mol­ecule is partly disordered (atoms N2, C13–C18) over two positions with occupancies of 0.544 (17) and 0.456 (17).

**Figure 2:**
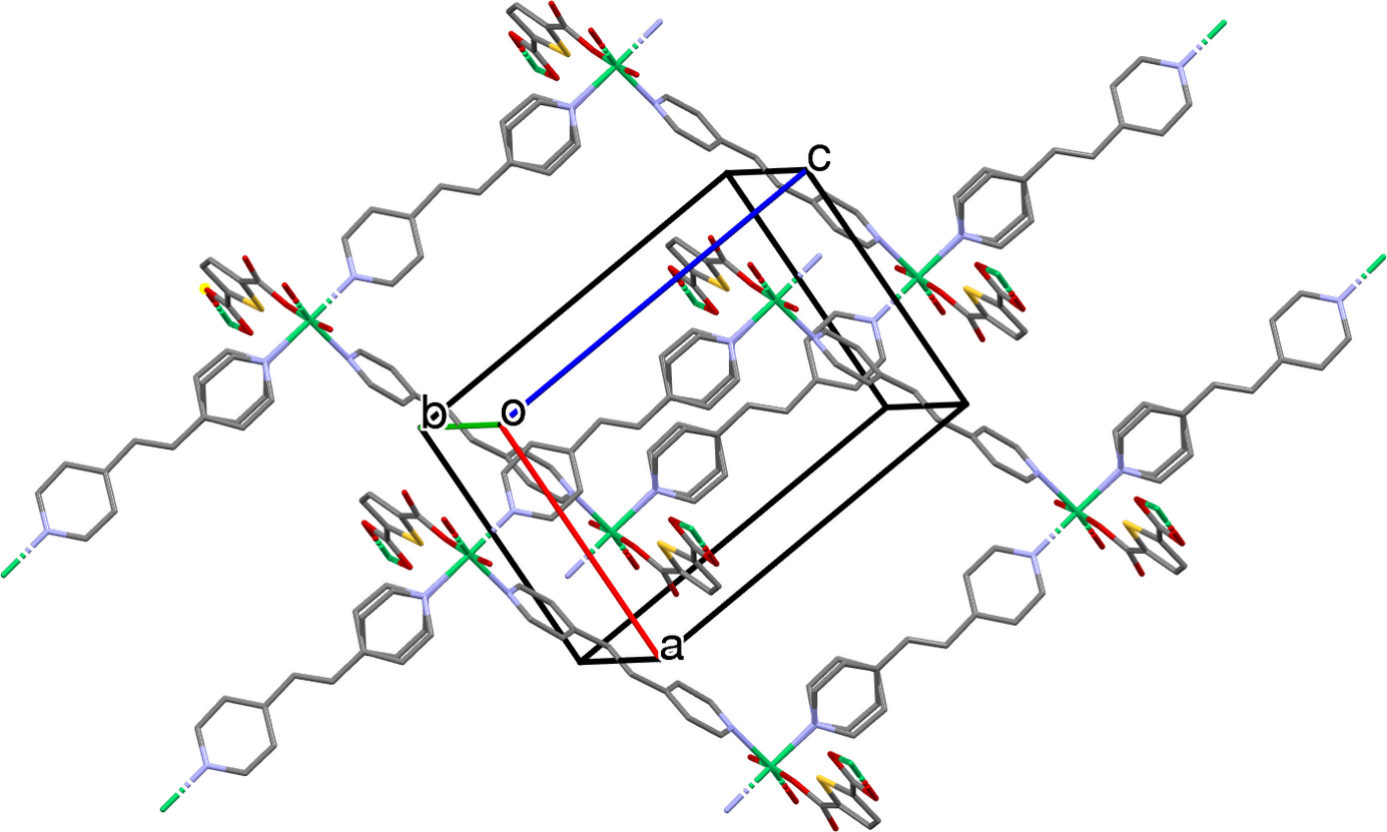
Packing diagram of Ni-HT-Bpe. Hydrogen atoms and DMF mol­ecules are omitted for clarity.

The Ni ion is octa­hedrally coordinated by three N atoms from Bpe [atoms N1, N2 and N3(*x*, *y*, −1 + *z*)] and three O atoms from HT [atoms O1, O3(*x*, 1 + *y*, *z*) and O4(*x*, 1 + *y*, *z*)] (Table 1 ▸). This results in chain formation in three directions: in the *c*-axis direction by inter­actions with N2 and N3, close to the *a*-axis direction by inter­actions with N1, and in the *b*-axis direction by inter­actions with O1, O3 and O4 (Table 1 ▸, Fig. 2 ▸). Oxygen atom O2 does not inter­act with the Ni ion, but forms a hydrogen bond with the neighbouring Bpe pyridyl ring (atom H8, see Table 2 ▸). Oxygen atoms O3 and O4 also show hydrogen bonds with the other Bpe mol­ecule (atoms H21 and H19*A*, respectively, see Table 2 ▸).

The DMF mol­ecule is positioned close to the disordered Bpe part and one of the methyl groups inter­acts with it through a C—H⋯π inter­action (Table 2 ▸). In addition, a C=O⋯π inter­action is observed with a neighbouring (N1,C17–C11) ring. Two additional C—H⋯π inter­actions are listed in Table 2 ▸.

**Figure 3:**
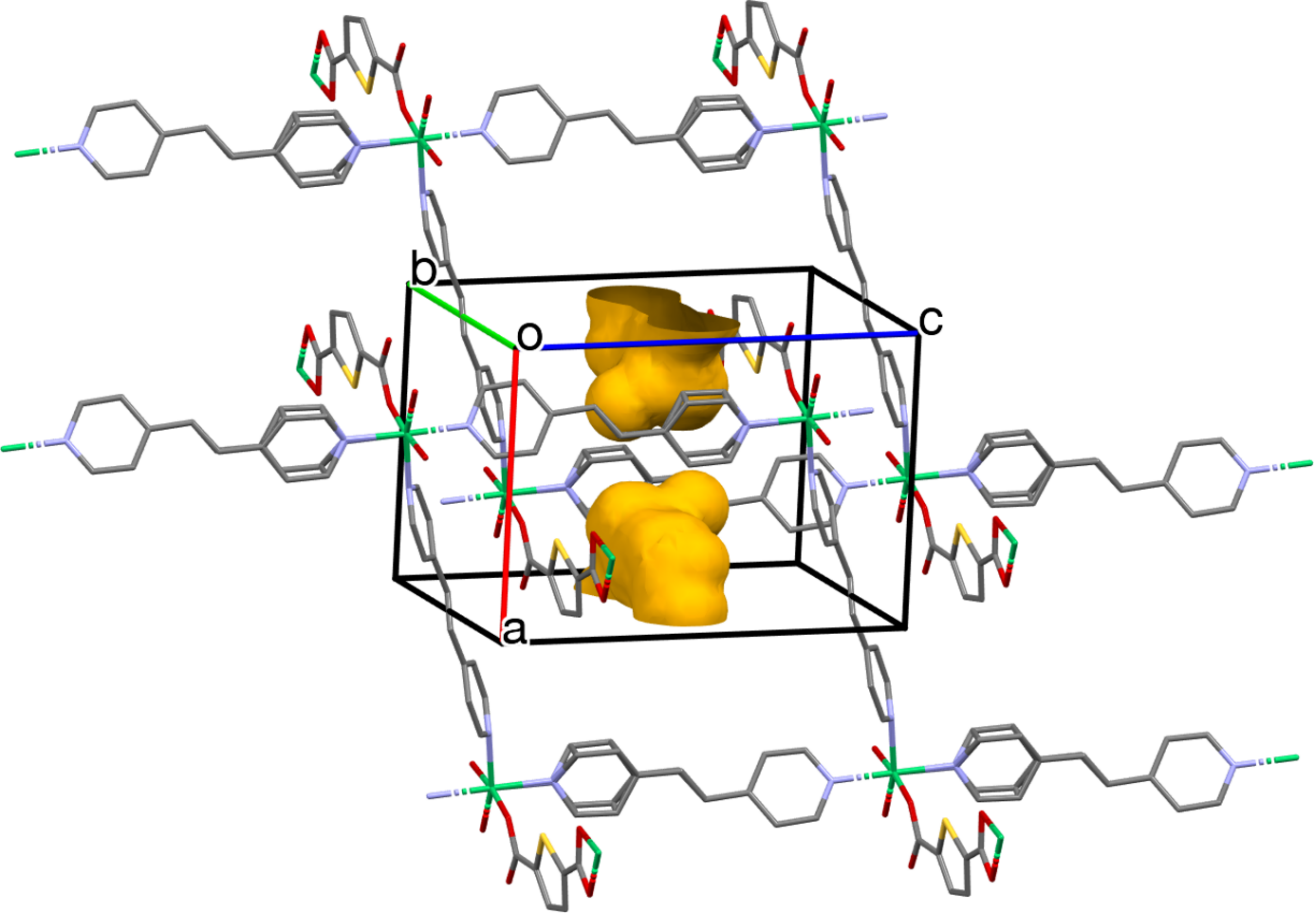
Visualization of the voids in the crystal packing of Ni-HT-Bpe using *Mercury* (Macrae *et al.*, 2020 ▸).

A void-space representation was generated using *Mercury* (Macrae *et al.*, 2020 ▸) for the solvent-free structural model (Fig. 3 ▸). The plot highlights the presence of inter­nal cavities within the unit cell, which appear as discrete void regions rather than a clearly continuous channel system. A qu­anti­tative porosity analysis was carried out using Zeo++ (Willems *et al.*, 2012 ▸) in high-accuracy mode using the solvent-free CIF. The framework exhibits a substantial geometric void volume of 454.2 Å^3^ per unit cell, corresponding to a void fraction of 0.372. However, the probe-accessible volume and accessible surface area are both zero for a probe radius of 1.86 Å (approximate the kinetic size of N_2_) indicating that the pore apertures are too small to be accessible to N_2_ under this probe condition.

The structure reveals that Ni nodes are bridged by HT and Bpe to generate an unusual two-dimensional layered framework, and the overall crystal is formed by an inter­locked stacking of these layers. Topological simplification classifies the framework as a non-inter­penetrated 3-nodal (2,2,5)-connected net, in which the Ni-containing node acts as the higher-connected vertex and the two organic ligands serve as 2-connected linkers propagating the connectivity within the layer. The experimental powder X-ray diffraction (PXRD) pattern is in good agreement with that simulated from the single-crystal structure, further confirming that the powder sample is consistent with the single-crystal model and exhibits good phase purity.

**Figure 4:**
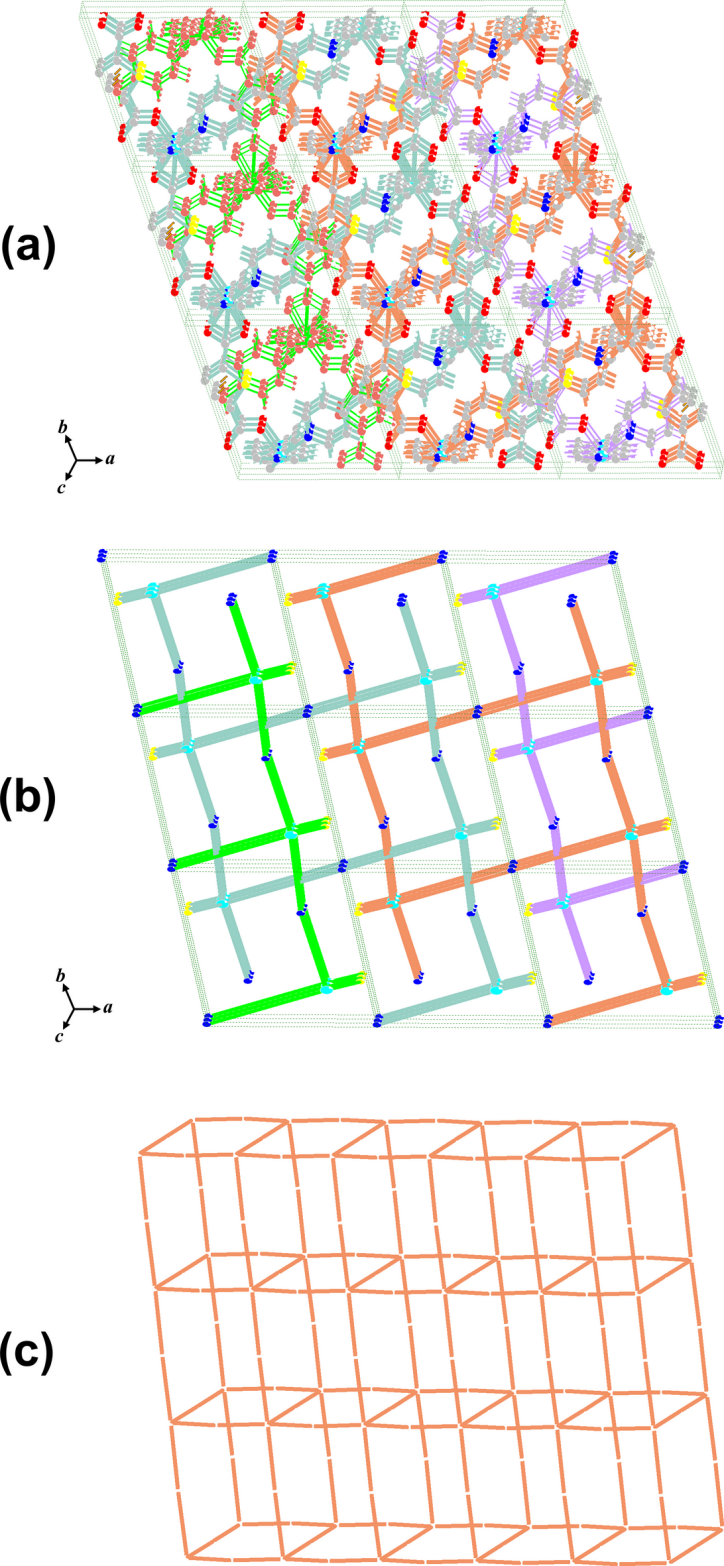
Topological analysis of Ni-HT-Bpe: (*a*) 3 × 3 × 3 unit cells of the framework; (*b*) 3 × 3 × 3 unit cells view of the corresponding simplified net; (*c*) representation of a single two-dimensional layer [parallel to (100)].

The connectivity of Ni-HT-Bpe was analyzed by a topological simplification in which the coordination framework is reduced to its underlying net (Fig. 4 ▸; Blatov *et al.*, 2014 ▸). The structure forms 2D layers parallel to (100), and only one structural group is present, indicating that the framework is non-inter­penetrated. In the reduced representation, the layer can be described as a 3-nodal (2,2,5)-connected net. The metal-containing node (originating from the Ni coordination environment) acts as the higher-connected vertex, while both organic linkers function as 2-connected spacers that propagate the network within the layer. The resulting topology is 2^2^,5-c net with stoichiometry (2-c)_4_(2-c)(5-c)_2_.

## 3. Database survey

A search of the Cambridge Structural Database (CSD, version 6.01, November 2025; Groom *et al.*, 2016 ▸) for thio­phene-2,5-di­carboxyl­ate resulted in 868 hits with 27 containing an O⋯Ni inter­action of which 20 are present in the MOF subset. A search for 1,2-bis­(pyridin-4-yl)ethyl­ene yielded 2868 hits with 123 showing an N⋯Ni inter­action of which 97 hits belong to the MOF subset.

Two structures are worthwhile to mention due to the presence of very similar building units. Refcode LICNER (Han *et al.*, 2007 ▸) refers to a Ni polymer containing thio­phene-2,5-di­carboxyl­ate (tda) and 1,3-di-pyridin-4-yl­propane (bpp). Each Ni ion is six-coordinated by four O atoms (two from two independent tda and two aqua O atoms) and two N atoms from two bpp ligands. A 2D grid-type bilayer formed through inter­molecular O—H⋯O inter­actions is running parallel to the (001) plane.

The asymmetric unit of the second one, KIFBOT (Lu *et al.*, 2018 ▸), contains one Ni atom, one thio­phene-2,5-di­carboxyl­ate anion (tdc), one 2,2′-dimethyl-4,4′-bi­pyridine ligand (dmbpy) and one μ_2_-O atom. The Ni ion is six-coordinated by three carboxyl­ate O atoms from three different tda, two N atoms from two different dmbpy and one μ_2_-O atom. A dimeric Ni unit [Ni_2_(COO)_4_(μ_2_-OH)] acts as secondary building unit (SBU) and neighbouring SBUs are connected by tdc ligands to form 2D grids, which extend into a 3D framework by dmbpy pillars.

## 4. Synthesis and crystallization

**Figure 5:**
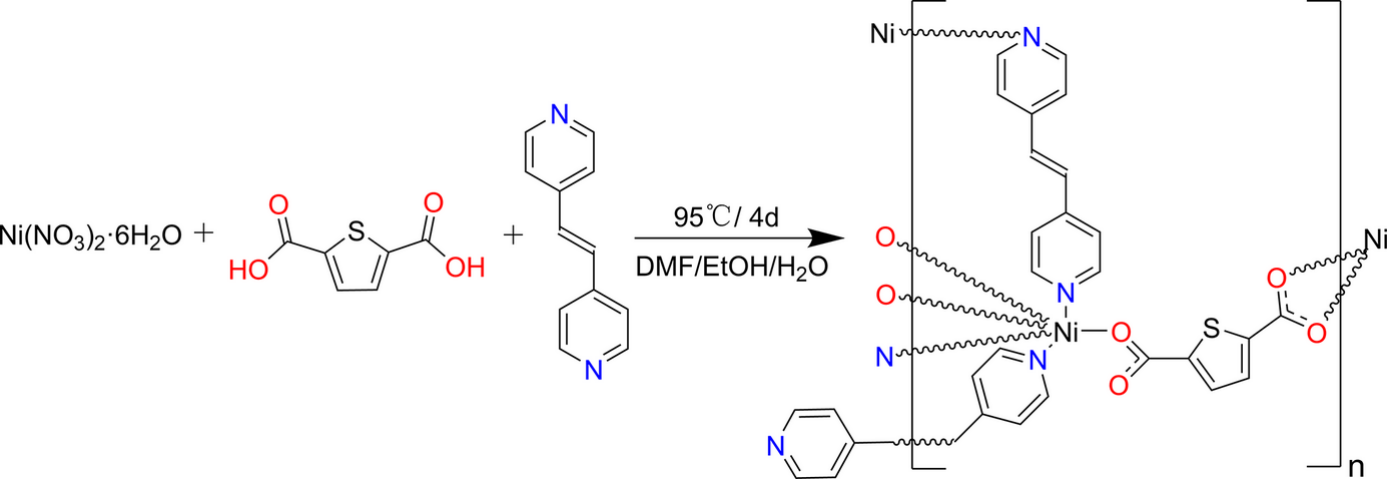
Reaction scheme for the synthesis of Ni-HT-Bpe.

The reaction scheme to synthesize the title compound is given in Fig. 5 ▸.

Ni(NO_3_)_2_·6H_2_O (29 mg, 0.10 mmol), thio­phene-2,5-di­carb­oxy­lic acid (HT; 15.6 mg, 0.10 mmol) and trans-1,2-bis­(pyridin-4-yl)ethyl­ene (Bpe; 27 mg, 0.15 mmol) were placed in a 25 mL Teflon-lined stainless-steel autoclave, and DMF/EtOH/H_2_O (10 mL) was added. The mixture was sonicated for 5 min and then stirred at room temperature for 10 min to give a homogeneous suspension. The vessel was sealed and heated at 368K for 4 days and then cooled to room temperature at a rate of 6 K h^−1^. Green block-shaped crystals and needle-like crystals of Ni-HT-Bpe were obtained. For powder preparation, the as-synthesized product was collected by filtration, washed with DMF (3 × 10 mL) followed by EtOH (3 × 10 mL), and dried in a vacuum oven at 353K overnight to afford Ni-HT-Bpe as green powder (54 mg, 75% yield based on Bpe).

**Figure 6:**
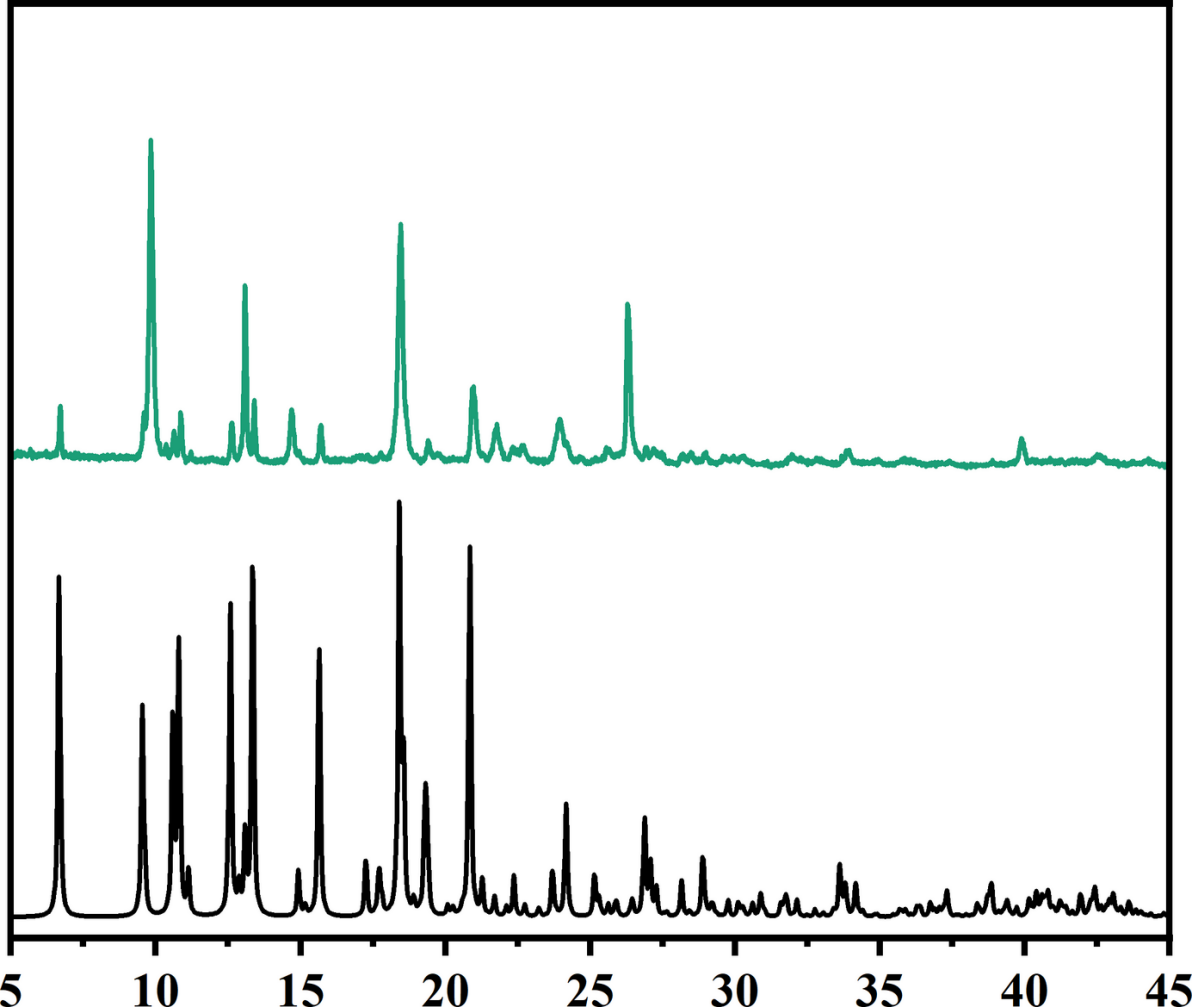
Experimental (top) and simulated (bottom) PXRD patterns of Ni-HT-Bpe. The experimental pattern was recorded using Cu *K*α radiation, and the simulated pattern was calculated using *Mercury* (Macrae *et al.*, 2020 ▸) based on the single-crystal structure.

**Figure 7:**
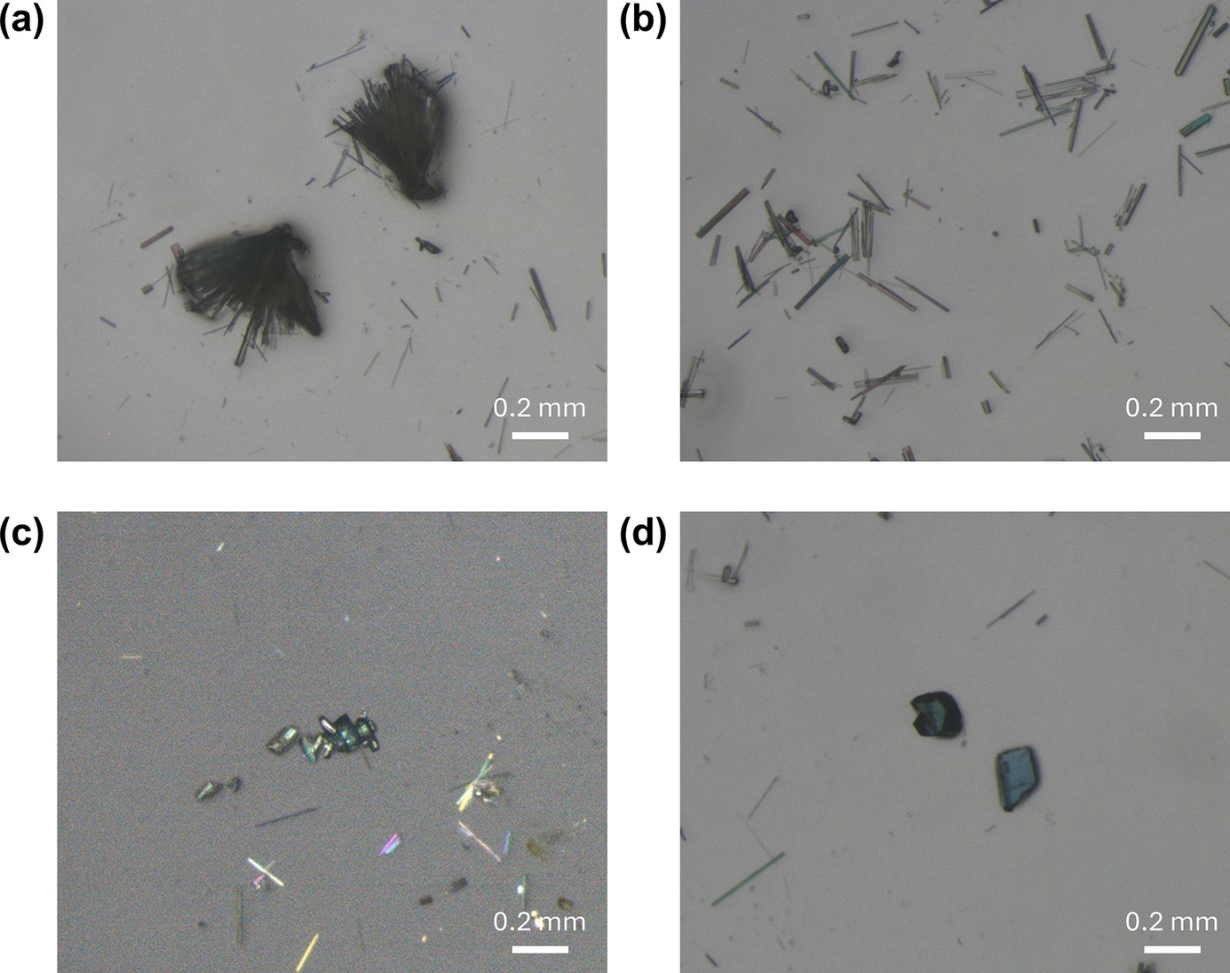
Optical micrographs of needle-like and block-like crystals in the as-synthesized sample (bright-field); panel (*c*) was acquired under crossed polarizers.

The phase purity of the powder sample was assessed by powder X-ray diffraction (PXRD). Powder X-ray diffraction data were collected on a PANalytical Empyrean diffractometer (Malvern Panalytical) in transmission geometry over the 2θ range 1.3–45°, using a PIXcel3D hybrid pixel detector and Cu *K*α radiation (*K*α_1_, λ = 1.5406 Å; *K*α_2_, λ = 1.5444 Å). The experimental PXRD pattern matches well with the peak positions in the pattern simulated from the single-crystal X-ray structure using *Mercury* (Macrae *et al.*, 2020 ▸), confirming that the crystalline powder material is consistent with the single-crystal model (Fig. 6 ▸). Noticeable discrepancies in relative intensities are observed. As the sample contains both needle-shaped and block-like crystals (Fig. 7 ▸), these discrepancies can be attributed mainly to preferred orientation effects arising from the strongly anisotropic, needle-shaped crystallites (and their inter­grown bundles) present in the bulk sample, whereas the simulated pattern assumes an ideal randomly oriented powder.

## 5. Refinement

Crystal data, data collection and structure refinement details are summarized in Table 3 ▸. Part of *trans*-1,2-bis­(pyrid­yl)ethyl­ene (atoms N2, C13-C18) was disordered over two positions with occupancies of 0.544 (17) and 0.456 (17). The DMF mol­ecule with refined occupancy 0.205 (7) was subject to DFIX, FLAT, RIGU and ISOR restraints to maintain the expected geometry.

## Supplementary Material

Crystal structure: contains datablock(s) I. DOI: 10.1107/S2056989026002276/vu2018sup1.cif

Structure factors: contains datablock(s) I. DOI: 10.1107/S2056989026002276/vu2018Isup2.hkl

CCDC reference: 2534227

Additional supporting information:  crystallographic information; 3D view; checkCIF report

## Figures and Tables

**Table 1:** Selected bond lengths (Å)

Ni1—O1	2.017 (3)	Ni1—N1	2.094 (3)
Ni1—O3^i^	2.156 (3)	Ni1—N3^ii^	2.112 (3)
Ni1—O4^i^	2.144 (3)	Ni1—N2	2.109 (9)

Symmetry codes: (i) 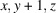; (ii) 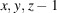.

**Table 2:** Hydrogen-bond geometry (Å, °) *Cg*1, *Cg*2 and *Cg*3 are the centroids of rings (N2*A*,C13*A*–C17*A*), (N3,C20–C24) and (N1,C7–C11), respectively.

*D*—H⋯*A*	*D*—H	H⋯*A*	*D*⋯*A*	*D*—H⋯*A*
C8—H8⋯O2^iii^	0.93	2.59	3.314 (7)	135
C19—H19A⋯O4^iv^	0.93	2.51	3.430 (6)	169
C21—H21⋯O3^v^	0.93	2.41	3.331 (6)	171
C27—H27*C*⋯*Cg*1	0.96	2.78	3.57 (6)	140
C10—H10⋯*Cg*2^v^	0.93	2.88	3.696 (6)	147
C22—H22⋯*Cg*3^vi^	0.93	2.93	3.534 (5)	124

Symmetry codes: (iii) 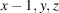; (iv) 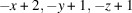; (v) 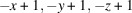; (vi) 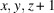.

**Table 3:** Experimental details

Crystal data	
Chemical formula	[Ni(C_6_H_3_O_4_S)(C_12_H_10_N_2_)_1.5_]·0.205C_3_H_7_NO
*M*_r_	517.08
Crystal system, space group	Triclinic, *P* 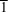
Temperature (K)	273
*a*, *b*, *c* (Å)	9.7666 (4), 9.8999 (4), 13.5669 (6)
α, β, γ (°)	98.092 (2), 96.235 (2), 107.823 (2)
*V* (Å^3^)	1220.25 (9)
*Z*	2
Radiation type	Cu *K*α
μ (mm^−1^)	2.26
Crystal size (mm)	0.15 × 0.13 × 0.12

Data collection	
Diffractometer	Bruker D8 Venture
Absorption correction	Multi-scan (*SADABS*; Krause *et al.,* 2015 ▸)
*T*_min_, *T*_max_	0.487, 0.754
No. of measured, independent and observed [*I* > 2σ(*I*)] reflections	16259, 4801, 3940
*R*_int_	0.058
(sin θ/λ)_max_ (Å^−1^)	0.620

Refinement	
*R*[*F*^2^ > 2σ(*F*^2^)], *wR*(*F*^2^), *S*	0.066, 0.185, 1.04
No. of reflections	4801
No. of parameters	411
No. of restraints	77
H-atom treatment	H-atom parameters constrained
Δρ_max_, Δρ_min_ (e Å^−3^)	0.43, −0.60

Computer programs: *SAINT* (Bruker, 2015 ▸), *SHELXT2018/2* (Sheldrick, 2015*a* ▸), *SHELXL2016/4* (Sheldrick, 2015*b* ▸) and *OLEX2* (Dolomanov *et al.*, 2009 ▸).

## supplementary crystallographic information

### Poly[[sesqui[µ-trans-1,2-bis(pyridin-4-yl)ethylene](µ-thiophene-2,5-dicarboxylato)nickel(II)] dimethylformamide 0.205-solvate] Crystal data

**Table:**

[Ni(C_6_H_3_O_4_S)(C_12_H_10_N_2_)_1.5_]·0.205C_3_H_7_NO	*Z* = 2
*M**_r_* = 517.08	*F*(000) = 532
Triclinic, *P*1	*D*_x_ = 1.407 Mg m^−^^3^
*a* = 9.7666 (4) Å	Cu *K*α radiation, λ = 1.54178 Å
*b* = 9.8999 (4) Å	Cell parameters from 8971 reflections
*c* = 13.5669 (6) Å	θ = 5.6–72.4°
α = 98.092 (2)°	µ = 2.26 mm^−^^1^
β = 96.235 (2)°	*T* = 273 K
γ = 107.823 (2)°	Block, clear light green
*V* = 1220.25 (9) Å^3^	0.15 × 0.13 × 0.12 mm

### Poly[[sesqui[µ-trans-1,2-bis(pyridin-4-yl)ethylene](µ-thiophene-2,5-dicarboxylato)nickel(II)] dimethylformamide 0.205-solvate] Data collection

**Table:**

Bruker D8 Venture diffractometer	3940 reflections with *I* > 2σ(*I*)
Detector resolution: 7.9 pixels mm^-1^	*R*_int_ = 0.058
φ and ω scans	θ_max_ = 73.0°, θ_min_ = 5.6°
Absorption correction: multi-scan (SADABS; Krause et al., 2015)	*h* = −10→12
*T*_min_ = 0.487, *T*_max_ = 0.754	*k* = −12→12
16259 measured reflections	*l* = −16→16
4801 independent reflections	

### Poly[[sesqui[µ-trans-1,2-bis(pyridin-4-yl)ethylene](µ-thiophene-2,5-dicarboxylato)nickel(II)] dimethylformamide 0.205-solvate] Refinement

**Table:**

Refinement on *F*^2^	Hydrogen site location: inferred from neighbouring sites
Least-squares matrix: full	H-atom parameters constrained
*R*[*F*^2^ > 2σ(*F*^2^)] = 0.066	*w* = 1/[σ^2^(*F*_o_^2^) + (0.0753*P*)^2^ + 2.321*P*] where *P* = (*F*_o_^2^ + 2*F*_c_^2^)/3
*wR*(*F*^2^) = 0.185	(Δ/σ)_max_ < 0.001
*S* = 1.04	Δρ_max_ = 0.43 e Å^−^^3^
4801 reflections	Δρ_min_ = −0.60 e Å^−^^3^
411 parameters	Extinction correction: SHELXL-2016/4 (Sheldrick, 2015b), Fc^*^=kFc[1+0.001xFc^2^λ^3^/sin(2θ)]^-1/4^
77 restraints	Extinction coefficient: 0.0106 (15)

### Poly[[sesqui[µ-trans-1,2-bis(pyridin-4-yl)ethylene](µ-thiophene-2,5-dicarboxylato)nickel(II)] dimethylformamide 0.205-solvate] Special details

**Table:**

Geometry. All esds (except the esd in the dihedral angle between two l.s. planes) are estimated using the full covariance matrix. The cell esds are taken into account individually in the estimation of esds in distances, angles and torsion angles; correlations between esds in cell parameters are only used when they are defined by crystal symmetry. An approximate (isotropic) treatment of cell esds is used for estimating esds involving l.s. planes.

### Poly[[sesqui[µ-trans-1,2-bis(pyridin-4-yl)ethylene](µ-thiophene-2,5-dicarboxylato)nickel(II)] dimethylformamide 0.205-solvate] Fractional atomic coordinates and isotropic or equivalent isotropic displacement parameters (Å^2^)

**Table:**

	*x*	*y*	*z*	*U*_iso_*/*U*_eq_	Occ. (<1)
Ni1	0.69283 (7)	0.86205 (6)	0.22629 (4)	0.0380 (3)	
S1	0.75169 (11)	0.38941 (11)	0.23040 (9)	0.0504 (3)	
O1	0.7559 (3)	0.6858 (3)	0.2229 (2)	0.0504 (7)	
O2	0.9965 (4)	0.7930 (3)	0.2616 (3)	0.0727 (10)	
O3	0.6566 (3)	0.0670 (3)	0.2320 (2)	0.0470 (6)	
O4	0.8783 (3)	0.0550 (3)	0.2622 (2)	0.0459 (6)	
N1	0.4762 (4)	0.7301 (4)	0.1770 (2)	0.0458 (8)	
N3	0.7129 (4)	0.8640 (3)	1.0730 (2)	0.0423 (7)	
C1	0.8847 (5)	0.6871 (4)	0.2486 (3)	0.0485 (9)	
C2	0.8999 (5)	0.5449 (4)	0.2600 (4)	0.0516 (10)	
C3	1.0229 (5)	0.5158 (5)	0.2889 (5)	0.0751 (16)	
H3	1.114325	0.585919	0.307182	0.090*	
C4	0.9981 (5)	0.3673 (5)	0.2886 (5)	0.0744 (16)	
H4	1.071606	0.329647	0.306474	0.089*	
C5	0.8571 (5)	0.2859 (4)	0.2596 (4)	0.0518 (10)	
C6	0.7934 (4)	0.1281 (4)	0.2508 (3)	0.0434 (8)	
C7	0.3659 (5)	0.7815 (5)	0.1773 (3)	0.0537 (10)	
H7	0.382073	0.872090	0.215811	0.064*	
C8	0.2282 (5)	0.7078 (6)	0.1236 (4)	0.0649 (12)	
H8	0.154093	0.748213	0.126837	0.078*	
C9	0.2014 (5)	0.5726 (5)	0.0647 (4)	0.0607 (12)	
C10	0.3152 (6)	0.5201 (6)	0.0651 (5)	0.0744 (15)	
H10	0.302092	0.430102	0.026884	0.089*	
C11	0.4482 (5)	0.5981 (5)	0.1210 (4)	0.0604 (12)	
H11	0.522886	0.558289	0.120386	0.072*	
C12	0.0596 (5)	0.4845 (6)	0.0014 (4)	0.0686 (13)	
H12	0.056538	0.399799	−0.039758	0.082*	
N2a	0.6832 (11)	0.8719 (19)	0.3816 (7)	0.049 (6)	0.544 (17)
C13a	0.5639 (11)	0.877 (2)	0.4220 (7)	0.074 (4)	0.544 (17)
H13a	0.484307	0.881302	0.379870	0.089*	0.544 (17)
C14a	0.5542 (11)	0.876 (2)	0.5208 (6)	0.080 (5)	0.544 (17)
H14a	0.465101	0.865300	0.542590	0.096*	0.544 (17)
C15a	0.6735 (16)	0.889 (2)	0.5887 (9)	0.051 (4)	0.544 (17)
C16a	0.8002 (12)	0.8912 (16)	0.5493 (8)	0.050 (3)	0.544 (17)
H16a	0.883435	0.894223	0.591251	0.060*	0.544 (17)
C17a	0.8004 (13)	0.8888 (16)	0.4472 (9)	0.053 (3)	0.544 (17)
H17a	0.888120	0.899723	0.423208	0.064*	0.544 (17)
C18a	0.660 (2)	0.879 (2)	0.6943 (10)	0.051 (5)	0.544 (17)
H18a	0.568903	0.867991	0.712312	0.062*	0.544 (17)
N12b	0.6761 (16)	0.867 (2)	0.3781 (12)	0.059 (9)	0.456 (17)
C113b	0.5687 (17)	0.762 (2)	0.4074 (8)	0.094 (7)	0.456 (17)
H113b	0.498886	0.693813	0.357604	0.113*	0.456 (17)
C114b	0.5589 (17)	0.752 (2)	0.5057 (8)	0.092 (7)	0.456 (17)
H114b	0.479659	0.682122	0.521112	0.111*	0.456 (17)
C115b	0.6630 (16)	0.841 (2)	0.5816 (9)	0.043 (4)	0.456 (17)
C116b	0.779 (2)	0.9444 (17)	0.5532 (10)	0.064 (4)	0.456 (17)
H116b	0.851599	1.011918	0.601356	0.076*	0.456 (17)
C117b	0.7812 (19)	0.9422 (19)	0.4510 (10)	0.063 (5)	0.456 (17)
H117b	0.865749	1.000082	0.432854	0.076*	0.456 (17)
C118b	0.653 (3)	0.832 (3)	0.6879 (11)	0.054 (6)	0.456 (17)
H118b	0.561079	0.786706	0.702699	0.065*	0.456 (17)
C19	0.7642 (5)	0.8832 (5)	0.7661 (3)	0.0506 (10)	
H19a	0.856178	0.892861	0.749447	0.061*	0.544 (17)
H19Ab	0.857626	0.926208	0.752932	0.061*	0.456 (17)
C20	0.7444 (4)	0.8739 (5)	0.8704 (3)	0.0468 (9)	
C21	0.6174 (5)	0.8778 (5)	0.9068 (3)	0.0517 (10)	
H21	0.539662	0.883691	0.863557	0.062*	
C22	0.6070 (5)	0.8729 (5)	1.0051 (3)	0.0512 (10)	
H22	0.520791	0.875931	1.026875	0.061*	
C23	0.8363 (4)	0.8605 (4)	1.0386 (3)	0.0462 (9)	
H23	0.912659	0.855557	1.083746	0.055*	
C24	0.8546 (5)	0.8638 (5)	0.9401 (3)	0.0486 (9)	
H24	0.941229	0.859288	0.919813	0.058*	
C25	0.532 (4)	0.489 (3)	0.613 (3)	0.115 (10)	0.205 (7)
H25	0.469078	0.488621	0.659763	0.138*	0.205 (7)
O5	0.653 (4)	0.486 (4)	0.638 (3)	0.158 (12)	0.205 (7)
N4	0.488 (4)	0.494 (4)	0.517 (3)	0.120 (9)	0.205 (7)
C26	0.339 (4)	0.500 (5)	0.496 (4)	0.124 (11)	0.205 (7)
H26A	0.285901	0.468594	0.548200	0.186*	0.205 (7)
H26B	0.290252	0.438990	0.432179	0.186*	0.205 (7)
H26C	0.343390	0.597964	0.492258	0.186*	0.205 (7)
C27	0.571 (6)	0.498 (6)	0.434 (4)	0.147 (15)	0.205 (7)
H27A	0.519568	0.419088	0.380124	0.221*	0.205 (7)
H27B	0.664506	0.490555	0.457324	0.221*	0.205 (7)
H27C	0.584640	0.587607	0.410528	0.221*	0.205 (7)

### Poly[[sesqui[µ-trans-1,2-bis(pyridin-4-yl)ethylene](µ-thiophene-2,5-dicarboxylato)nickel(II)] dimethylformamide 0.205-solvate] Atomic displacement parameters (Å^2^)

**Table:**

	*U*^11^	*U*^22^	*U*^33^	*U*^12^	*U*^13^	*U*^23^
Ni1	0.0410 (4)	0.0368 (4)	0.0366 (4)	0.0120 (3)	0.0031 (2)	0.0118 (2)
S1	0.0459 (6)	0.0397 (5)	0.0669 (7)	0.0161 (4)	0.0007 (4)	0.0156 (4)
O1	0.0501 (16)	0.0438 (15)	0.0586 (17)	0.0175 (12)	0.0002 (13)	0.0154 (12)
O2	0.0541 (19)	0.0419 (17)	0.122 (3)	0.0139 (15)	0.0082 (19)	0.0239 (18)
O3	0.0405 (15)	0.0428 (14)	0.0574 (16)	0.0143 (12)	0.0012 (12)	0.0118 (12)
O4	0.0429 (14)	0.0370 (13)	0.0607 (17)	0.0163 (11)	0.0027 (12)	0.0152 (12)
N1	0.0434 (17)	0.0473 (18)	0.0438 (17)	0.0093 (14)	0.0025 (14)	0.0148 (14)
N3	0.0473 (18)	0.0440 (17)	0.0391 (16)	0.0176 (14)	0.0055 (13)	0.0139 (13)
C1	0.053 (2)	0.041 (2)	0.053 (2)	0.0175 (18)	0.0045 (18)	0.0138 (17)
C2	0.051 (2)	0.041 (2)	0.066 (3)	0.0169 (18)	0.0047 (19)	0.0168 (18)
C3	0.049 (3)	0.043 (2)	0.129 (5)	0.011 (2)	−0.005 (3)	0.027 (3)
C4	0.052 (3)	0.044 (2)	0.127 (5)	0.019 (2)	−0.005 (3)	0.024 (3)
C5	0.049 (2)	0.036 (2)	0.072 (3)	0.0162 (17)	0.004 (2)	0.0154 (18)
C6	0.045 (2)	0.0387 (19)	0.047 (2)	0.0141 (16)	0.0049 (16)	0.0112 (16)
C7	0.048 (2)	0.060 (3)	0.049 (2)	0.015 (2)	0.0038 (18)	0.0068 (19)
C8	0.048 (2)	0.077 (3)	0.070 (3)	0.021 (2)	0.004 (2)	0.018 (3)
C9	0.055 (3)	0.059 (3)	0.055 (3)	0.006 (2)	−0.007 (2)	0.008 (2)
C10	0.062 (3)	0.057 (3)	0.089 (4)	0.012 (2)	−0.010 (3)	−0.001 (3)
C11	0.049 (2)	0.045 (2)	0.076 (3)	0.0095 (19)	−0.006 (2)	0.002 (2)
C12	0.055 (3)	0.066 (3)	0.075 (3)	0.014 (2)	−0.003 (2)	0.004 (2)
N2a	0.038 (9)	0.088 (12)	0.020 (6)	0.021 (7)	0.006 (5)	0.004 (6)
C13a	0.059 (6)	0.134 (13)	0.042 (5)	0.047 (7)	0.005 (4)	0.022 (6)
C14a	0.051 (5)	0.158 (15)	0.042 (5)	0.047 (7)	0.013 (4)	0.023 (6)
C15a	0.055 (7)	0.056 (11)	0.038 (5)	0.017 (6)	0.002 (4)	0.007 (5)
C16a	0.044 (5)	0.072 (8)	0.039 (5)	0.024 (5)	0.005 (3)	0.016 (5)
C17a	0.044 (5)	0.075 (9)	0.044 (5)	0.022 (5)	0.008 (4)	0.019 (5)
C18a	0.048 (7)	0.062 (12)	0.047 (6)	0.022 (7)	0.011 (4)	0.010 (5)
N12b	0.060 (15)	0.046 (10)	0.068 (14)	0.009 (8)	0.001 (10)	0.028 (8)
C113b	0.082 (9)	0.111 (14)	0.041 (6)	−0.032 (9)	−0.007 (6)	0.016 (7)
C114b	0.082 (9)	0.108 (14)	0.047 (6)	−0.027 (9)	−0.006 (6)	0.027 (7)
C115b	0.048 (7)	0.050 (11)	0.030 (6)	0.012 (5)	0.006 (4)	0.011 (5)
C116b	0.071 (10)	0.063 (9)	0.044 (6)	0.008 (7)	−0.002 (6)	0.009 (6)
C117b	0.058 (8)	0.075 (11)	0.046 (7)	0.001 (7)	0.011 (6)	0.021 (7)
C118b	0.062 (8)	0.064 (15)	0.031 (6)	0.007 (9)	0.009 (5)	0.017 (6)
C19	0.052 (2)	0.064 (3)	0.038 (2)	0.020 (2)	0.0086 (17)	0.0131 (18)
C20	0.048 (2)	0.052 (2)	0.040 (2)	0.0163 (18)	0.0068 (16)	0.0120 (17)
C21	0.049 (2)	0.070 (3)	0.042 (2)	0.025 (2)	0.0068 (17)	0.0194 (19)
C22	0.049 (2)	0.069 (3)	0.045 (2)	0.027 (2)	0.0090 (17)	0.0205 (19)
C23	0.044 (2)	0.051 (2)	0.043 (2)	0.0150 (17)	0.0024 (16)	0.0128 (17)
C24	0.047 (2)	0.058 (2)	0.042 (2)	0.0177 (19)	0.0074 (17)	0.0123 (18)
C25	0.121 (12)	0.103 (14)	0.120 (12)	0.033 (9)	0.022 (8)	0.019 (9)
O5	0.135 (14)	0.16 (2)	0.167 (19)	0.046 (14)	0.010 (12)	0.026 (15)
N4	0.125 (12)	0.109 (13)	0.118 (11)	0.028 (9)	0.024 (7)	0.013 (9)
C26	0.134 (14)	0.103 (19)	0.124 (19)	0.027 (15)	0.008 (12)	0.020 (16)
C27	0.16 (2)	0.14 (2)	0.139 (18)	0.033 (16)	0.046 (16)	0.030 (16)

### Poly[[sesqui[µ-trans-1,2-bis(pyridin-4-yl)ethylene](µ-thiophene-2,5-dicarboxylato)nickel(II)] dimethylformamide 0.205-solvate] Geometric parameters (Å, º)

**Table:**

Ni1—O1	2.017 (3)	C14a—H14a	0.9300
Ni1—O3^i^	2.156 (3)	C14a—C15a	1.364 (13)
Ni1—O4^i^	2.144 (3)	C15a—C16a	1.396 (14)
Ni1—N1	2.094 (3)	C15a—C18a	1.467 (11)
Ni1—N3^ii^	2.112 (3)	C16a—H16a	0.9300
Ni1—C6^i^	2.472 (4)	C16a—C17a	1.383 (11)
Ni1—N2	2.109 (9)	C17a—H17a	0.9300
Ni1—N12b	2.079 (15)	C18a—H18a	0.9300
S1—C2	1.721 (4)	C18a—C19	1.31 (2)
S1—C5	1.714 (4)	N12b—C113b	1.371 (14)
O1—C1	1.264 (5)	N12b—C117b	1.300 (14)
O2—C1	1.236 (5)	C113b—H113b	0.9300
O3—Ni1^iii^	2.156 (3)	C113b—C114b	1.363 (12)
O3—C6	1.266 (5)	C114b—H114b	0.9300
O4—Ni1^iii^	2.144 (3)	C114b—C115b	1.359 (14)
O4—C6	1.268 (5)	C115b—C116b	1.401 (14)
N1—C7	1.325 (6)	C115b—C118b	1.470 (11)
N1—C11	1.347 (6)	C116b—H116b	0.9300
N3—Ni1^iv^	2.112 (3)	C116b—C117b	1.387 (13)
N3—C22	1.341 (5)	C117b—H117b	0.9300
N3—C23	1.347 (5)	C118b—H118b	0.9300
C1—C2	1.488 (5)	C118b—C19	1.35 (2)
C2—C3	1.349 (6)	C19—H19a	0.9300
C3—H3	0.9300	C19—H19Ab	0.9300
C3—C4	1.414 (6)	C19—C20	1.461 (5)
C4—H4	0.9300	C20—C21	1.393 (6)
C4—C5	1.347 (6)	C20—C24	1.391 (6)
C5—C6	1.476 (5)	C21—H21	0.9300
C6—Ni1^iii^	2.472 (4)	C21—C22	1.355 (6)
C7—H7	0.9300	C22—H22	0.9300
C7—C8	1.381 (6)	C23—H23	0.9300
C8—H8	0.9300	C23—C24	1.370 (5)
C8—C9	1.393 (7)	C24—H24	0.9300
C9—C10	1.363 (7)	C25—H25	0.9300
C9—C12	1.486 (6)	C25—O5	1.213 (19)
C10—H10	0.9300	C25—N4	1.34 (2)
C10—C11	1.366 (7)	N4—C26	1.47 (2)
C11—H11	0.9300	N4—C27	1.46 (2)
C12—C12^v^	1.290 (10)	C26—H26A	0.9600
C12—H12	0.9300	C26—H26B	0.9600
N2a—C13a	1.352 (8)	C26—H26C	0.9600
N2a—C17a	1.323 (8)	C27—H27A	0.9600
C13a—H13a	0.9300	C27—H27B	0.9600
C13a—C14a	1.355 (11)	C27—H27C	0.9600
			
O1—Ni1—O3^i^	172.16 (11)	N2a—C13a—H13a	118.3
O1—Ni1—O4^i^	110.62 (11)	N2a—C13a—C14a	123.4 (8)
O1—Ni1—N1	90.21 (13)	C14a—C13a—H13a	118.3
O1—Ni1—N3^ii^	91.18 (12)	C13a—C14a—H14a	119.6
O1—Ni1—C6^i^	141.40 (13)	C13a—C14a—C15a	120.8 (9)
O1—Ni1—N2a	90.2 (5)	C15a—C14a—H14a	119.6
O1—Ni1—N12b	90.1 (5)	C14a—C15a—C16a	116.4 (10)
O3^i^—Ni1—C6^i^	30.80 (12)	C14a—C15a—C18a	120.5 (13)
O4^i^—Ni1—O3^i^	61.62 (10)	C16a—C15a—C18a	122.4 (13)
O4^i^—Ni1—C6^i^	30.85 (11)	C15a—C16a—H16a	120.4
N1—Ni1—O3^i^	97.44 (12)	C17a—C16a—C15a	119.1 (9)
N1—Ni1—O4^i^	158.61 (12)	C17a—C16a—H16a	120.4
N1—Ni1—N3^ii^	87.44 (13)	N2a—C17a—C16a	123.9 (10)
N1—Ni1—C6^i^	127.99 (13)	N2a—C17a—H17a	118.1
N1—Ni1—N2a	95.7 (3)	C16a—C17a—H17a	118.1
N3^ii^—Ni1—O3^i^	87.47 (12)	C15a—C18a—H18a	116.6
N3^ii^—Ni1—O4^i^	87.32 (12)	C19—C18a—C15a	126.8 (16)
N3^ii^—Ni1—C6^i^	85.91 (13)	C19—C18a—H18a	116.6
N2a—Ni1—O3^i^	90.7 (5)	C113b—N12b—Ni1	120.4 (11)
N2a—Ni1—O4^i^	89.2 (4)	C117b—N12b—Ni1	123.5 (10)
N2a—Ni1—N3^ii^	176.6 (4)	C117b—N12b—C113b	113.9 (12)
N2a—Ni1—C6^i^	91.0 (5)	N12b—C113b—H113b	118.3
N12b—Ni1—O3^i^	91.1 (5)	C114b—C113b—N12b	123.4 (11)
N12b—Ni1—O4^i^	91.1 (4)	C114b—C113b—H113b	118.3
N12b—Ni1—N1	93.8 (4)	C113b—C114b—H114b	119.5
N12b—Ni1—N3^ii^	178.3 (5)	C115b—C114b—C113b	121.0 (11)
N12b—Ni1—C6^i^	92.4 (5)	C115b—C114b—H114b	119.5
C5—S1—C2	91.8 (2)	C114b—C115b—C116b	116.6 (11)
C1—O1—Ni1	125.3 (3)	C114b—C115b—C118b	121.4 (14)
C6—O3—Ni1^iii^	88.5 (2)	C116b—C115b—C118b	122.0 (13)
C6—O4—Ni1^iii^	89.0 (2)	C115b—C116b—H116b	121.1
C7—N1—Ni1	122.3 (3)	C117b—C116b—C115b	117.9 (12)
C7—N1—C11	116.5 (4)	C117b—C116b—H116b	121.1
C11—N1—Ni1	119.3 (3)	N12b—C117b—C116b	126.0 (13)
C22—N3—Ni1^iv^	122.7 (3)	N12b—C117b—H117b	117.0
C22—N3—C23	116.0 (3)	C116b—C117b—H117b	117.0
C23—N3—Ni1^iv^	121.2 (3)	C115b—C118b—H118b	116.9
O1—C1—C2	115.4 (4)	C19—C118b—C115b	126.3 (19)
O2—C1—O1	126.2 (4)	C19—C118b—H118b	116.9
O2—C1—C2	118.3 (4)	C18a—C19—H19a	117.9
C1—C2—S1	121.0 (3)	C18a—C19—C20	124.2 (8)
C3—C2—S1	111.0 (3)	C118b—C19—H19Ab	118.5
C3—C2—C1	127.9 (4)	C118b—C19—C20	123.0 (9)
C2—C3—H3	123.6	C20—C19—H19a	117.9
C2—C3—C4	112.9 (4)	C20—C19—H19Ab	118.5
C4—C3—H3	123.6	C21—C20—C19	122.8 (4)
C3—C4—H4	123.4	C24—C20—C19	121.2 (4)
C5—C4—C3	113.1 (4)	C24—C20—C21	116.0 (4)
C5—C4—H4	123.4	C20—C21—H21	119.9
C4—C5—S1	111.2 (3)	C22—C21—C20	120.1 (4)
C4—C5—C6	127.3 (4)	C22—C21—H21	119.9
C6—C5—S1	121.4 (3)	N3—C22—C21	124.3 (4)
O3—C6—Ni1^iii^	60.67 (19)	N3—C22—H22	117.8
O3—C6—O4	120.7 (3)	C21—C22—H22	117.8
O3—C6—C5	120.6 (3)	N3—C23—H23	118.5
O4—C6—Ni1^iii^	60.12 (19)	N3—C23—C24	123.1 (4)
O4—C6—C5	118.7 (4)	C24—C23—H23	118.5
C5—C6—Ni1^iii^	176.5 (3)	C20—C24—H24	119.8
N1—C7—H7	118.2	C23—C24—C20	120.5 (4)
N1—C7—C8	123.5 (4)	C23—C24—H24	119.8
C8—C7—H7	118.2	O5—C25—H25	120.0
C7—C8—H8	120.3	O5—C25—N4	120 (4)
C7—C8—C9	119.4 (5)	N4—C25—H25	120.0
C9—C8—H8	120.3	C25—N4—C26	115 (3)
C8—C9—C12	124.6 (5)	C25—N4—C27	127 (4)
C10—C9—C8	116.7 (4)	C27—N4—C26	118 (4)
C10—C9—C12	118.7 (5)	N4—C26—H26A	109.5
C9—C10—H10	119.6	N4—C26—H26B	109.5
C9—C10—C11	120.8 (5)	N4—C26—H26C	109.5
C11—C10—H10	119.6	H26A—C26—H26B	109.5
N1—C11—C10	123.1 (5)	H26A—C26—H26C	109.5
N1—C11—H11	118.4	H26B—C26—H26C	109.5
C10—C11—H11	118.4	N4—C27—H27A	109.5
C9—C12—H12	117.2	N4—C27—H27B	109.5
C12^v^—C12—C9	125.6 (7)	N4—C27—H27C	109.5
C12^v^—C12—H12	117.2	H27A—C27—H27B	109.5
C13a—N2a—Ni1	123.1 (8)	H27A—C27—H27C	109.5
C17a—N2a—Ni1	121.1 (7)	H27B—C27—H27C	109.5
C17a—N2a—C13a	115.5 (9)		
			
Ni1—O1—C1—O2	−14.6 (7)	C10—C9—C12—C12^v^	175.1 (8)
Ni1—O1—C1—C2	167.8 (3)	C11—N1—C7—C8	−0.5 (7)
Ni1^iii^—O3—C6—O4	−3.5 (4)	C12—C9—C10—C11	179.0 (5)
Ni1^iii^—O3—C6—C5	176.2 (4)	N2a—C13a—C14a—C15a	9 (3)
Ni1^iii^—O4—C6—O3	3.6 (4)	C13a—N2a—C17a—C16a	9 (2)
Ni1^iii^—O4—C6—C5	−176.2 (4)	C13a—C14a—C15a—C16a	−5 (3)
Ni1—N1—C7—C8	163.5 (4)	C13a—C14a—C15a—C18a	−176.3 (15)
Ni1—N1—C11—C10	−163.1 (4)	C14a—C15a—C16a—C17a	4 (2)
Ni1^iv^—N3—C22—C21	178.4 (4)	C14a—C15a—C18a—C19	176.7 (16)
Ni1^iv^—N3—C23—C24	−179.0 (3)	C15a—C16a—C17a—N2a	−6 (2)
Ni1—N2a—C13a—C14a	176.4 (12)	C15a—C18a—C19—C20	179.3 (13)
Ni1—N2a—C17a—C16a	−177.7 (11)	C16a—C15a—C18a—C19	6 (3)
Ni1—N12b—C113b—C114b	174.4 (16)	C17a—N2a—C13a—C14a	−10 (2)
Ni1—N12b—C117b—C116b	−176.8 (14)	C18a—C15a—C16a—C17a	174.6 (14)
S1—C2—C3—C4	−0.7 (7)	C18a—C19—C20—C21	−8.4 (13)
S1—C5—C6—O3	−7.8 (6)	C18a—C19—C20—C24	173.0 (11)
S1—C5—C6—O4	172.0 (3)	N12b—C113b—C114b—C115b	−5 (3)
O1—C1—C2—S1	4.6 (6)	C113b—N12b—C117b—C116b	−13 (3)
O1—C1—C2—C3	−178.1 (5)	C113b—C114b—C115b—C116b	1 (3)
O2—C1—C2—S1	−173.2 (4)	C113b—C114b—C115b—C118b	180 (2)
O2—C1—C2—C3	4.1 (8)	C114b—C115b—C116b—C117b	−3 (3)
N1—C7—C8—C9	−0.6 (8)	C114b—C115b—C118b—C19	160 (2)
N3—C23—C24—C20	1.2 (7)	C115b—C116b—C117b—N12b	10 (3)
C1—C2—C3—C4	−178.2 (5)	C115b—C118b—C19—C20	177.9 (16)
C2—S1—C5—C4	−1.1 (5)	C116b—C115b—C118b—C19	−21 (3)
C2—S1—C5—C6	179.7 (4)	C117b—N12b—C113b—C114b	10 (3)
C2—C3—C4—C5	−0.1 (8)	C118b—C115b—C116b—C117b	178.2 (19)
C3—C4—C5—S1	0.9 (7)	C118b—C19—C20—C21	−31.1 (15)
C3—C4—C5—C6	180.0 (5)	C118b—C19—C20—C24	150.4 (14)
C4—C5—C6—O3	173.2 (5)	C19—C20—C21—C22	−178.1 (4)
C4—C5—C6—O4	−7.1 (8)	C19—C20—C24—C23	177.6 (4)
C5—S1—C2—C1	178.7 (4)	C20—C21—C22—N3	−0.2 (7)
C5—S1—C2—C3	1.0 (5)	C21—C20—C24—C23	−1.0 (6)
C7—N1—C11—C10	1.3 (7)	C22—N3—C23—C24	−0.8 (6)
C7—C8—C9—C10	0.9 (8)	C23—N3—C22—C21	0.3 (7)
C7—C8—C9—C12	−178.2 (5)	C24—C20—C21—C22	0.5 (7)
C8—C9—C10—C11	−0.1 (8)	O5—C25—N4—C26	−179 (3)
C8—C9—C12—C12^v^	−5.8 (11)	O5—C25—N4—C27	−1 (3)
C9—C10—C11—N1	−1.1 (9)		

Symmetry codes: (i) *x*, *y*+1, *z*; (ii) *x*, *y*, *z*−1; (iii) *x*, *y*−1, *z*; (iv) *x*, *y*, *z*+1; (v) −*x*, −*y*+1, −*z*.

### Poly[[sesqui[µ-trans-1,2-bis(pyridin-4-yl)ethylene](µ-thiophene-2,5-dicarboxylato)nickel(II)] dimethylformamide 0.205-solvate] Hydrogen-bond geometry (Å, º)

*Cg*1, *Cg*2 and *Cg*3 are the centroids of rings (N2*A*,C13*A*–C17*A*), (N3,C20–C24) and (N1,C7–C11), respectively.

**Table:**

*D*—H···*A*	*D*—H	H···*A*	*D*···*A*	*D*—H···*A*
C8—H8···O2^vi^	0.93	2.59	3.314 (7)	135
C19—H19A···O4^vii^	0.93	2.51	3.430 (6)	169
C21—H21···O3^viii^	0.93	2.41	3.331 (6)	171
C27—H27*C*···*Cg*1	0.96	2.78	3.57 (6)	140
C10—H10···*Cg*2^viii^	0.93	2.88	3.696 (6)	147
C22—H22···*Cg*3^iv^	0.93	2.93	3.534 (5)	124

Symmetry codes: (iv) *x*, *y*, *z*+1; (vi) *x*−1, *y*, *z*; (vii) −*x*+2, −*y*+1, −*z*+1; (viii) −*x*+1, −*y*+1, −*z*+1.
